# Long-term safety and efficacy of adjunctive rasagiline in levodopa-treated Japanese patients with Parkinson’s disease

**DOI:** 10.1007/s00702-018-1962-5

**Published:** 2019-01-11

**Authors:** Nobutaka Hattori, Atsushi Takeda, Shinichi Takeda, Akira Nishimura, Ryou Nakaya, Hideki Mochizuki, Masahiro Nagai, Ryosuke Takahashi

**Affiliations:** 1 Department of Neurology, Juntendo University Faculty of Medicine, 2-1-1, Hongo, Bunkyo-ku, Tokyo, 113-8421 Japan; 2 Department of Neurology, National Hospital Organization, Sendai Nishitaga Hospital, Sendai, Miyagi Japan; 3 Japan Development Center, Takeda Pharmaceutical Company Limited, Osaka, Japan; 4 Department of Neurology, Osaka University Graduate School of Medicine, Osaka, Japan; 5 Clinical Therapeutic Trial Center, Ehime University Hospital, Toon, Ehime Japan; 6 Department of Neurology, Kyoto University Graduate School of Medicine, Kyoto, Japan

**Keywords:** Rasagiline, Japanese, Adjunctive therapy, Parkinson’s disease, Wearing-off

## Abstract

Rasagiline is a monoamine oxidase type-B inhibitor in development in Japan for Parkinson’s disease (PD). This open-label study evaluated the long-term safety and efficacy of rasagiline in Japanese patients with PD receiving levodopa. Patients were aged 30–79 years and had wearing-off or weakened effect. Patients received rasagiline 1 mg/day for 52 weeks. The primary objective was to evaluate safety. Secondary endpoints included MDS-UPDRS Part II and Part III total scores (ON-state) and change from baseline in mean daily OFF-time. An additional endpoint was the Parkinson’s Disease Questionnaire-39 (PDQ-39) Summary Index (SI) score. In total, 222 patients were enrolled; 52.3% had wearing-off phenomena. Treatment-emergent adverse events (TEAEs) were mostly mild or moderate and occurred in 83.3% of patients; 63.1% had drug-related TEAEs; and 21.2% had TEAEs resulting in discontinuation. Fall (16.7%), nasopharyngitis (14.0%), and dyskinesia (10.8%) were the most frequent TEAEs. Serious TEAEs were reported in 17.6% of patients, and led to discontinuation in 9.5%. At week 52 (last-observation-carried forward), the mean change from baseline in MDS-UPDRS Part III total score (ON-state) was − 7.6; the mean change from baseline in daily OFF-time was − 0.89 h in patients with wearing-off phenomena at the start of the run-in period. The mean change from baseline in PDQ-39 SI was − 0.64. No major safety issues were observed during this 52-week trial of rasagiline as an adjunct to levodopa in Japanese patients. Mean changes in MDS-UPDRS scores and daily OFF-time suggested that adjunctive rasagiline treatment with levodopa was efficacious, with efficacy maintained for at least 52 weeks.

## Introduction

Parkinson’s disease (PD) is a common neurodegenerative disease, that is second only in prevalence to Alzheimer’s disease and affects more than 300 per 100,000 individuals worldwide, most of whom are older than 50 years (Giovannoni et al. 2016; Pagano et al. 2016; Pringsheim et al. 2014). In Japan, 100–150 per 100,000 people suffer from the condition (Giovannoni et al. 2016), the prevalence of which is expected to increase, in parallel with the anticipated increase in the proportion of older people in the population.

Although the etiology of PD is complex, the key pathological feature is an early loss of dopaminergic neurons and reduced levels of dopamine in the nigrostriatal system (Kalia and Lang 2015). This is believed to be the main underlying cause of the characteristic motor symptoms of the condition, namely resting tremor, muscle rigidity, bradykinesia, and postural instability. Hence, most approved PD drugs [including levodopa, dopamine agonists, catechol-*O*-methyl transferase (COMT) inhibitors, and monoamine oxidase-B inhibitors (MAOB-Is)] are thought to alleviate symptoms by enhancing levels of dopamine or inhibiting its metabolism in specific brain regions. Drugs that do not act directly on the dopaminergic system (and which are less commonly used therapeutically) include anticholinergic drugs and amantadine (Schapira and Olanow 2008).

Levodopa is the naturally occurring immediate precursor of dopamine and is the most effective and commonly used drug for treating the motor symptoms of PD (Kalia and Lang 2015; Schapira 2007; Schapira and Olanow 2008; Tsouli and Konitsiotis 2010). However, long-term use can lead to motor complications, including wearing-off phenomena, which occur when the duration of therapeutic benefit of each levodopa dose gradually declines (Bhidayasiri et al. 2015; Chen et al. 2014; Schapira and Olanow 2008). Approximately 45% of Asian patients with PD develop wearing-off phenomena (Chen et al. 2014; Yoritaka et al. 2013). Japanese and international guidelines recommend that wearing-off is managed by adjusting the dose and/or formulation of levodopa or by use of adjunctive pharmacotherapy such as MAOB-Is (e.g., selegiline, rasagiline) and COMT inhibitors (Chen et al. 2014; DeMaagd and Philip 2015; Ferreira et al. 2013; Stowe et al. 2011; Takeda 2013). In Japan, istradefylline or zonisamide are also used adjunctively to levodopa for the management of wearing-off (Mizuno et al. 2010, 2013; Murata et al. 2007, 2015).

Rasagiline is a second-generation selective MAOB-I (Lecht et al. 2007) that is used both as monotherapy and in combination therapy in the treatment of PD (Chang et al. 2017). Based on findings from two phase 3 studies in patients with motor fluctuations during treatment with levodopa (Parkinson Study Group 2005; Rascol et al. 2005), rasagiline is currently indicated outside Japan as an adjunctive therapy for PD (Mitchell et al. 2005; Torkildsen et al. 2016).

We recently reported the results of two randomized, double-blind, placebo-controlled, 26-week studies of rasagiline therapy in Japanese patients with PD (Hattori et al. 2017, 2018). In combination with levodopa, rasagiline (0.5 or 1.0 mg/day) led to a reduction in mean daily OFF-time and an improvement in PD symptoms [assessed using the Movement Disorder Society-Unified Parkinson’s Disease Rating Scale (MDS-UPDRS)] (Kashihara et al. 2014) in Japanese patients with PD and wearing-off phenomena (Hattori et al. 2018). Furthermore, an improvement in quality of life [assessed using the Parkinson’s Disease Questionnaire (PDQ-39)] was observed and no new safety concerns were reported.

As PD is a chronic disease, long-term safety and efficacy data for rasagiline and levodopa combination therapy may provide useful insights for clinical practice. The two pivotal studies that evaluated the use of adjunctive rasagiline were 18- and 26-week studies (Parkinson Study Group 2005; Rascol et al. 2005). To our knowledge, no clinical studies have yet examined the long-term (> 26 weeks) safety and efficacy of rasagiline as an adjunctive treatment to levodopa in patients with PD. In the current study, we evaluated the safety and efficacy of long-term use of rasagiline 1 mg/day in combination with levodopa for up to 52 weeks in Japanese patients with PD.

## Methods

### Study design

This was a multi-center, open-label, phase 3 study designed to evaluate the safety and efficacy of long-term use of rasagiline (1 mg/day) as adjunctive therapy to levodopa, in Japanese patients with PD. The study began with a 2-week run-in period during which eligibility was determined. At the end of the run-in period, eligible patients received rasagiline 1 mg/day for up to 52 weeks. In principle, the dose of levodopa was to remain fixed from the start of the run-in period and throughout the treatment period. Change in levodopa dose was permitted, as deemed necessary by the investigators, but discontinuation was not allowed. Concomitant treatment with other PD agents (except selegiline) was permitted, provided that the dose did not change during the treatment period, wherever possible. Concomitant use of any antidepressants was prohibited. Patients used a diary to record ON-time without troublesome dyskinesia, ON-time with troublesome dyskinesia, OFF-time, and sleeping time, in 30-min intervals over 24 h during the 7 days preceding the weeks 0, 6, 10, 18, 26, 34, 42, and 52 visits.

The study was reviewed and approved by the Institutional Review Board at each of the participating study centers and was conducted in full compliance with the International Conference on Harmonization unified guidelines, the regulatory requirements of the region, and the ethical principles that have their origin in the Declaration of Helsinki. All patients provided written, informed consent. This study was registered with Clinical Trials.gov as NCT02337764.

### Patients

Adult patients aged between 30 and 79 years, with a diagnosis of PD according to the UK Parkinson’s Disease Society Brain Bank diagnostic criteria were eligible for inclusion in the study. All patients had been continuously receiving levodopa plus a dopa decarboxylase inhibitor for at least 1 month prior to the start of the run-in period and were experiencing wearing-off phenomena or weakened effect (defined as initial efficacy at the start of therapy, with diminished effect over time) during treatment with levodopa. Exclusion criteria included: a modified Hoehn and Yahr stage (Goetz et al. 2004) of 5 (at either ON-time or OFF-time for patients with wearing-off phenomena); severe dyskinesia or unstable systemic disease; a Mini-Mental State Examination score of ≤ 24 at the start of the run-in period; and neurosurgical intervention for PD.

### Endpoints and assessments

The primary endpoint was the incidence of treatment-emergent adverse events (TEAEs) during the 52-week treatment period. TEAEs were classified according to the Medical Dictionary for Regulatory Activities (MedDRA) version 19.0 and were defined as any adverse events occurring after the first administration of rasagiline. Secondary endpoints were the MDS-UPDRS Part II (motor aspects of experiences of daily living) and Part III (motor examination) (Kashihara et al. 2014) total scores during ON-state, and mean daily OFF-time (averaged over 7 days before each visit) for patients with wearing-off phenomena at the start of the run-in period. Additional endpoints included MDS-UPDRS Part I (non-motor aspects of experiences of daily living) and Part IV (motor complications) total scores, and the PDQ-39 summary index and individual domain scores. MDS-UPDRS evaluations were performed by qualified and accredited investigators (Part IA, Part III and Part IV) or by patient self-assessment (Part IB and Part II); lower MDS-UPDRS scores indicate milder PD symptoms/signs (Kashihara et al. 2014). PDQ-39 is a patient self-reported questionnaire that assesses quality of life; lower scores indicate better quality of life (Jenkinson et al. 1997).

Patients were evaluated at weeks 3, 6, 10, 18, 26, 34, 42, and 52 during the treatment period. MDS-UPDRS scores were determined at weeks 0 and 6 and at every visit thereafter; PDQ-39 scores were determined at weeks 0, 26, and 52; TEAEs were recorded at every visit.

### Statistical analysis

The safety analysis included data from patients who received at least one dose of the study drug. The last-observation-carried forward (LOCF) method was used to impute missing values for the 52-week timepoint for secondary efficacy endpoints and the additional endpoint of PDQ-39 scores. Descriptive statistics [means and two-sided 95% confidence intervals (CI)] were calculated for the changes from baseline in MDS-UPDRS total scores and mean daily OFF-time (for patients who had wearing-off phenomena during the run-in period) at each clinic visit. Similarly, descriptive statistics (means and two-sided 95% CI) were calculated for PDQ-39 scores. Taking feasibility into account, the planned number of patients to receive the study drug was set at 215.

## Results

### Patients

**Fig. 1:**
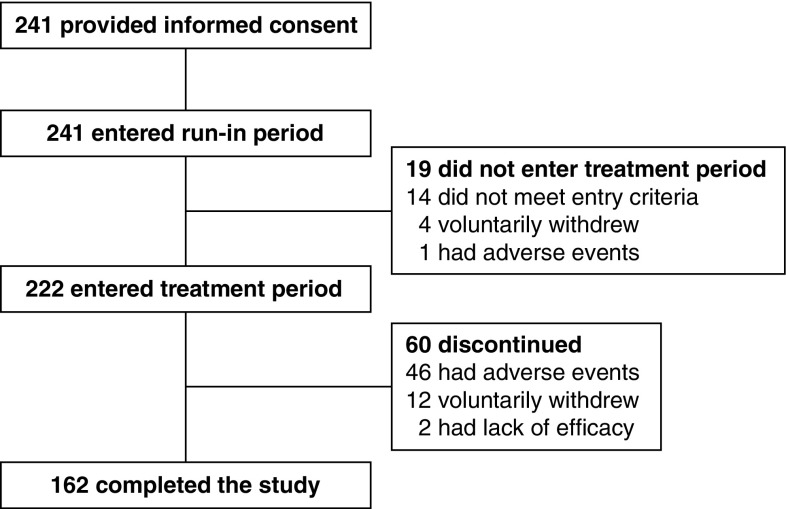
Patient disposition

Of 241 patients providing informed consent, 222 patients were eligible to enter the study. Sixty patients (27%) discontinued before the end of the study; reasons for withdrawal were TEAEs (46 patients), voluntary withdrawal (12 patients), and lack of efficacy (2 patients) (Fig. 1).

At baseline (start of treatment), the mean age was 68 years, and the mean duration of PD was approximately 7 years (Table 1). The proportion of patients requiring a dose of levodopa of more than 300 mg/day was 77.8%. The mean duration of levodopa administration was 4.61 years. The proportion of patients with wearing-off phenomena was 52.3% with mean duration of wearing-off phenomena of approximately 3 years. The average MDS-UPDRS Part III (ON-state), II, I, and IV total scores at baseline were 28.8, 11.9, 7.8, and 3.0, respectively. Dopamine agonists were the most commonly used concomitant therapy for PD (Table 1).

**Table 1:** Baseline demographics and clinical characteristics

	Rasagiline 1 mg (*n* = 222)
Age, years, mean (SD)	68.0 (8.41)
Age ≥ 65 years, *n* (%)	168 (75.7)
Male gender, *n* (%)	97 (43.7)
Duration of PD, years, mean (SD)	7.09 (5.022)
Duration of PD ≥ 10 years, *n* (%)	42 (18.9)
MDS-UPDRS Part I score, mean (SD)	7.8 (4.72)
MDS-UPDRS Part II score, mean (SD)	11.9 (7.28)
MDS-UPDRS Part III score (ON-state), mean (SD)	28.8 (13.14)
MDS-UPDRS Part IV score, mean (SD)	3.0 (3.61)
PDQ-39 Summary Index score, mean (SD)	18.06 (12.121)
Patients with wearing-off phenomena, *n* (%)	116 (52.3)
Duration of wearing-off phenomena, years, mean (SD)	3.02 (2.747)
Modified Hoehn and Yahr stage, mean (SD)	
ON-state	2.47 (0.688)
OFF-state	3.19 (0.700)
Daily OFF-time, hours, mean (SD)	4.99 (3.263)
Patients without wearing-off phenomena, *n* (%)	106 (47.7)
Modified Hoehn and Yahr stage, mean (SD)	2.42 (0.708)
Duration of levodopa use, years, mean (SD)	4.61 (4.217)
Levodopa total daily dose, mg, mean (SD)	355.0 (147.11)
Levodopa frequency per day; mean (SD)	3.3 (1.00)
Concomitant therapy, *n* (%)	
Dopamine agonists	163 (73.4)
COMT inhibitors	75 (33.8)
Amantadine	54 (24.3)
Anticholinergics	36 (16.2)
Zonisamide	36 (16.2)
Istradefylline	26 (11.7)
Droxidopa	15 (6.8)

*COMT* catechol-*O*-methyl transferase, *MDS-UPDRS* Movement Disorder Society-Unified Parkinson’s Disease Rating Scale, *PDQ* Parkinson’s Disease Questionnaire, *SD* standard deviation

### Safety and tolerability

During the 52-week treatment period, 185 (83.3%) patients reported 599 TEAEs, and most of which were classified as mild to moderate (Table 2). Forty-seven patients (21.2%) discontinued the study due to TEAEs. Fall (16.7%), nasopharyngitis (14.0%), and dyskinesia (10.8%) were the most frequently occurring TEAEs (Table 3). The incidence of serious TEAEs was 17.6%; 9.5% of patients discontinued due to TEAEs (Table 2). During days 1–83 of rasagiline treatment, 116/222 patients (52.3%) had TEAEs, compared with 99/196 patients (50.5%) during days 84–167, 69/177 patients (39.0%) during days 168–251, 50/170 patients (29.4%) during days 252–335, and 19/164 patients (11.6%) after day 336. There were no deaths during the study.

**Table 2:** Summary of TEAEs

	Rasagiline 1 mg/day (*n* = 222)	
	Number of events	Number of patients (%)
Any TEAE	599	185 (83.3)
Treatment-related	310	140 (63.1)
Leading to study discontinuation	54	47 (21.2)
Severity		
Mild	477	108 (48.6)
Moderate	109	65 (29.3)
Severe	13	12 (5.4)
Serious TEAEs	47	39 (17.6)
Treatment-related	24	21 (9.5)
Leading to study discontinuation	22	21 (9.5)
Deaths	0	0

*TEAE* treatment-emergent adverse event

**Table 3:** TEAEs with overall incidence > 3%

*n* (%)	Rasagiline 1 mg/day (*n* = 222)
Fall	37 (16.7)
Nasopharyngitis	31 (14.0)
Dyskinesia	24 (10.8)
Contusion	19 (8.6)
Orthostatic hypotension	12 (5.4)
Headache	10 (4.5)
Decreased appetite	9 (4.1)
Dental caries	9 (4.1)
Eczema	9 (4.1)
Blood creatine phosphokinase increased	7 (3.2)
Constipation	7 (3.2)
Dehydration	7 (3.2)
Hallucination	7 (3.2)
Hypertension	7 (3.2)

*TEAE* treatment-emergent adverse event

### Efficacy

**Fig. 2:**
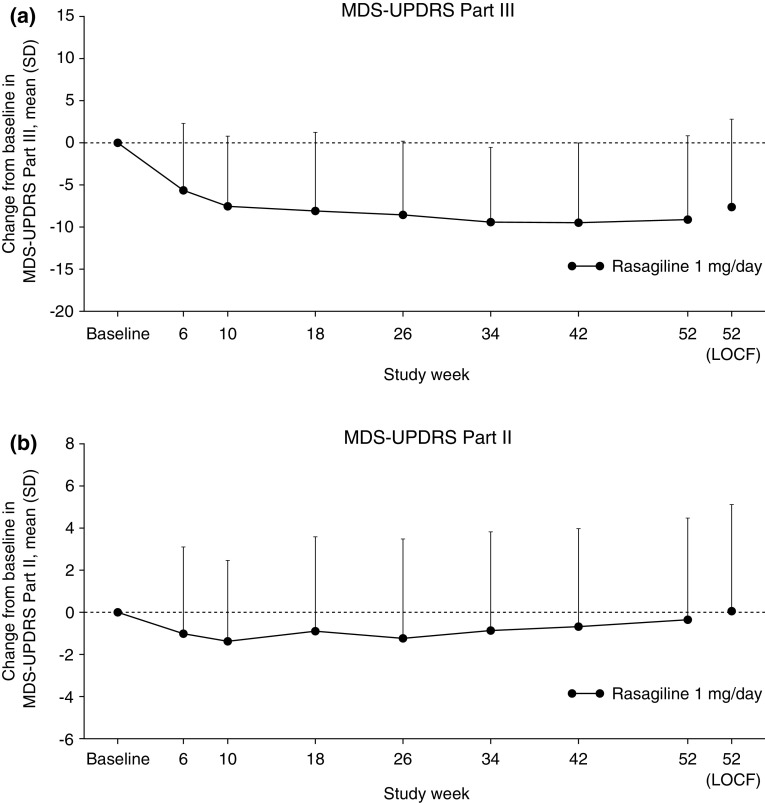
Mean change from baseline to week 52 in MDS-UPDRS Part III (ON-state) (**a**) and Part II (**b**) scores. *LOCF* last-observation carried forward, *MDS-UPDRS* Movement Disorder Society-Unified Parkinson’s Disease Rating Scale, *SD* standard deviation

After 6 weeks of adjunctive rasagiline, there was a greater than 5-point mean reduction in MDS-UPDRS Part III total score (ON-state); the improvement in score was maintained throughout the study (Fig. 2a). The mean change in MDS-UPDRS Part III total score (ON-state) from baseline to week 52 (LOCF) was − 7.6 (Table 4). By the first study visit (week 6), there was an approximate 1-point mean reduction in MDS-UPDRS Part II total score, followed by a small mean increase in score over the remaining weeks of the study (Fig. 2b). The mean change in MDS-UPDRS Part II total score from baseline to week 52 (LOCF) was 0.0 (Table 4). For MDS-UPDRS Part I and Part IV total scores, the mean changes from baseline to week 52 (LOCF) were 0.3 and − 0.2, respectively (Table 4).

**Table 4:** Changes in MDS-UPDRS scores, daily OFF-time and PDQ-39 scores from baseline to week 52 (LOCF)

	Change in score, mean (SD)	95% CI	
		Lower limit	Upper limit
MDS-UPDRS Part I score	0.3 (4.15)	− 0.29	0.82
MDS-UPDRS Part II score	0.0 (5.08)	− 0.64	0.72
MDS-UPDRS Part III (ON-time) score	− 7.6 (10.45)	− 8.99	− 6.18
MDS-UPDRS Part IV score	− 0.2 (2.18)	− 0.44	0.14
Mean daily OFF-time	− 0.89 (2.537)	− 1.376	− 0.399
PDQ-39 Summary Index score	− 0.64 (9.413)	− 1.908	0.634
Mobility	− 0.97 (17.953)	− 3.399	1.451
Activities of daily living	− 2.21 (17.212)	− 4.535	0.114
Emotional well-being	0.37 (14.835)	− 1.630	2.378
Stigma	− 1.59 (12.251)	− 3.241	0.068
Social support	0.31 (11.646)	− 1.258	1.887
Cognitions	0.03 (16.377)	− 2.183	2.241
Communication	0.27 (13.887)	− 1.602	2.149
Bodily discomfort	− 1.29 (19.446)	− 3.917	1.336

*CI* confidence interval, *LOCF* last-observation carried forward, *MDS-UPDRS* Movement Disorder Society-Unified Parkinson’s Disease Rating Scale, *PDQ* Parkinson’s Disease Questionnaire, *SD* standard deviation

**Fig. 3:**
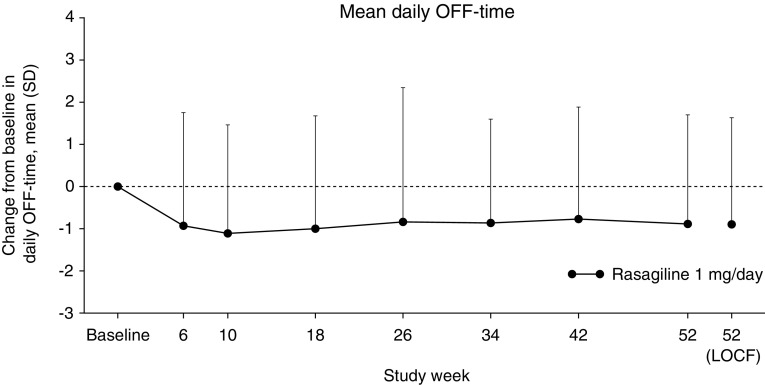
Mean change in daily OFF-time from baseline to week 52. *LOCF* last-observation carried forward, *SD* standard deviation

Among 116 patients with wearing-off phenomena at the start of run-in period, the mean daily OFF-time at the end of the run-in period was 4.99 h (Table 1). After 6 weeks of adjunctive rasagiline, there was a mean reduction in daily OFF-time of approximately 1 h (Fig. 3). The reduction was maintained throughout the treatment period. The mean change in daily OFF-time per day from baseline to week 52 (LOCF) was − 0.89 h (Table 4).

At baseline, the mean PDQ-39 summary index was 18.06; after 52 weeks (LOCF) of adjunctive rasagiline, the mean change from baseline in Summary Index score was − 0.64 points. Mean scores for individual PDQ-39 domains were as follows: mobility [29.82 at baseline, change from baseline to week 52 (LOCF), − 0.97]; activities of daily living (22.79, − 2.21); emotional well-being (18.33, 0.37); stigma (14.02, − 1.59); social support (6.26, 0.31); cognition (21.94, 0.03); communication (10.41, 0.27); bodily discomfort (20.85, − 1.29) (Table 4).

## Discussion

This single-arm, open-label, phase 3 study is the first evaluation of the safety and efficacy of long-term rasagiline as adjunctive therapy in patients with PD who had wearing-off phenomena or weakened effect during treatment with levodopa. TEAEs were generally mild to moderate, in line with the findings of the previous studies of rasagiline (Parkinson Study Group 2002, 2005; Rascol et al. 2005). Fall and nasopharyngitis were the most frequently occurring TEAEs. While these TEAEs were not reported as frequently in the PRESTO study or the LARGO study (Parkinson Study Group 2005; Rascol et al. 2005), the same TEAEs were reported in a 26-week study that investigated use of adjunctive rasagiline therapy in Japan (Hattori et al. 2018), as well as in other studies in Western populations (Olanow et al. 2009). In the PRESTO study, dyskinesia was reported more frequently in patients taking rasagiline 1 mg/day than in those taking 0.5 mg/day (Parkinson Study Group 2005). In the present study, the overall incidence of TEAEs was higher (52.3%) during days 1–83 after initiation of rasagiline than during subsequent periods; the incidence decreased over time. Administration of rasagiline for 52 weeks did not raise safety concerns in comparison with our previous study of adjunctive rasagiline for 26 weeks (Hattori et al. 2018). We highlight, however, that the rate of discontinuation due to TEAEs was 21.2% overall; as 18% had discontinued within the first 26 weeks, which aligns with results of the previous work [15.5% by week 26; (Hattori et al. 2018)], we attribute this seemingly high discontinuation rate to the long observation period in the present study.

The efficacy of long-term rasagiline in combination with levodopa was a secondary endpoint. To our knowledge, this was the first clinical trial to investigate the efficacy of 52 weeks of adjunctive rasagiline treatment in patients with PD, using MDS-UPDRS scores rather than the conventional UPDRS score. Treatment with adjunctive rasagiline led to improvements in motor function, as indicated by the sustained improvement in MDS-UPDRS Part III total score (ON-state). The mean improvement of − 7.6 points in MDS-UPDRS Part III total score (ON-state) from baseline to week 52 (LOCF) was considered to be clinically meaningful, based on the criteria proposed by Horvath et al. (Horvath et al. 2015). In patients with wearing-off phenomena at the start of the run-in period, treatment with rasagiline 1 mg/day led to a reduction in daily OFF-time versus baseline, which was maintained throughout the study; the mean change in daily OFF-time from baseline to week 52 (LOCF) was − 0.89 h. In our previous 26-week study, a greater mean decrease in daily OFF-time from baseline was observed in patients treated with rasagiline 1 mg/day (− 1.35) (Hattori et al. 2018). This difference between studies may be explained by the lack of an inclusion criterion in the present study defining daily OFF-time at baseline, which resulted in the enrollment of patients with shorter daily OFF-time in the present study relatively to the previous 26-week study (Hattori et al. 2018). Indeed, > 10% of patients with wearing-off phenomena had a daily OFF-time at baseline of less than 1 h, and in these patients, the change from baseline was understandably small, which may have affected the overall mean values. The present study was not designed to evaluate onset of efficacy in terms of reduction of daily OFF-time; however, a reduction was observed at the time of the first evaluation (6 weeks), similarly to our 26-week study and the PRESTO study (Hattori et al. 2018; Parkinson Study Group 2005).

Interpretations of the findings of this study are limited by the trial’s uncontrolled design. In the absence of a placebo arm, it is difficult to estimate the magnitude of effect that was attributable to the combination therapy. However, the mean changes from baseline at week 26 for the MDS-UPDRS Part III total score (ON-state) and daily OFF-time were similar to those determined in our previous study, in which there were significant differences versus the placebo group (Hattori et al. 2018), suggesting that the effects identified in the present study were drug-related. Since PD is a chronic disease and treatment is a life-long process, the present 52-week open-label study may not be sufficient to understand the long-term safety and efficacy of adjunctive rasagiline. Further longer term studies with treatment for > 52 weeks may be needed to advance our knowledge of this therapeutic strategy and optimize its clinical use.

In conclusion, rasagiline appears safe and tolerable as an adjunctive therapy in levodopa-treated Japanese patients with PD. Changes in MDS-UPDRS scores and daily OFF-time suggested that rasagiline treatment with levodopa was efficacious, with efficacy maintained for at least 52 weeks. These results support the use of rasagiline as a potential treatment option for Japanese patients with PD who develop wearing-off phenomena or weakened effect during treatment with levodopa.
